# Relationship between muscle and subcutaneous adipose tissue size and density and proximal femur bone in elderly women with hip fracture

**DOI:** 10.1007/s40520-024-02782-y

**Published:** 2024-06-11

**Authors:** Yangtong Li, Chenjun Liu, Jing Lu, Hui Sun, Yuehua Li

**Affiliations:** 1 Institute of Diagnostic and Interventional Radiology, Shanghai Sixth People’s Hospital Affiliated to Shanghai Jiao Tong University School of Medicine, Shanghai, 200233 China; 2 National Center for Orthopaedics, Shanghai Sixth People’s Hospital, Shanghai, 200233 China; 3 Department of Orthopaedic Surgery, Shanghai Sixth People’s Hospital Affiliated to Shanghai Jiao Tong University School of Medicine, Shanghai, 200233 China

**Keywords:** Hip fracture, Osteoporosis, Muscle, Subcutaneous adipose tissue, Bone mineral density

## Abstract

**Background:**

Both osteoporosis and sarcopenia are associated with aging, increasing the likelihood of falls in older adults and consequently raising the risk of hip fractures (HF).

**Aims:**

To explore the relationship between the size and density of muscle and subcutaneous adipose tissue (SAT) and the bone mineral density (BMD) of the proximal femur in elderly women with HF.

**Methods:**

Quantitative computed tomography (QCT) was conducted on the hips of 661 female participants who experienced low-energy acute HFs to measure both areal BMD (aBMD) and volume BMD (vBMD). Measurements were taken for the cross-sectional area (CSA) and density of the muscle around the hip and adjacent SAT. Multivariable linear regression models were applied to assess the relationship between these parameters.

**Results:**

Most increases in the density of the gluteus medius and minimus muscle (G.Med/MinM) were correlated with higher BMD in the femoral neck fracture (FNF) group with osteoporosis. In the FNF group, gluteus maximus muscle (G.MaxM) density was negatively associated with the BMD parameters of the proximal femur in individuals with osteoporosis, while they were positively associated with nonosteoporosis. In the intertrochanteric fracture (ITF) group without osteoporosis, both FN aBMD and FN vBMD showed significant correlations with G.Med/MinM density.

**Discussion:**

In women with HFs, bone and muscle are closely related.

**Conclusions:**

In older women with HFs, density but not CSA of the G.Med/MinM were associated with BMD parameters of the proximal femur. Osteoporosis may influence the relationship between G.MaxM density and proximal femur BMD in elderly women with FNF.

**Supplementary Information:**

The online version contains supplementary material available at 10.1007/s40520-024-02782-y.

## Introduction

Both osteoporosis and sarcopenia are associated with aging, increasing the likelihood of falls in older adults and consequently raising the risk of hip fractures (HF) [1–3]. HF is closely associated with low bone mineral density (BMD), increased repair costs, and a higher rate of disability and mortality compared to other types of osteoporotic fractures [2]. Osteoporosis is a chronic metabolic bone disease characterized by decreased BMD and deterioration of bony microarchitecture [4], while sarcopenia is a progressive and systemic skeletal muscle disease characterized by accelerated loss of muscle mass and function [5, 6]. Bone and muscle are two deeply interconnected organs with integrated functions in growth and locomotion [7]. A strong relationship exists between their development, maintenance and correct functioning. Muscle and bone interactions take place at different levels (organ, cellular, and molecular level) with bidirectional pathways [8]. There are several lines of evidence that support the concept that muscle and bone interact beyond the mechanical. For example, muscle flaps appear to accelerate bone healing after injury [9]. New data suggests that bone and muscle are also linked through biochemical communication through the musculoskeletal secretome [10]. In addition, both osteoblasts and adipocytes originate from a common progenitor and bone marrow skeletal stem cells multipotent mesenchymal stromal cells [11]. Secretion from adipocytes may have both negative and positive effects on bone [12]. Although close ties exist between their embryogenesis, growth and aging, the relationship between impairment of muscle, subcutaneous adipose tissue (SAT) and bone function remains unclear.

Quantitative computed tomography (QCT) utilizes 3D imaging to independently assess cortical and trabecular BMD [13], as well as anatomical muscle and adipose tissue [14]. More recently, several studies using QCT have demonstrated an association between lower lumbar BMD and poorer muscle quality [15, 16]. In healthy elderly adults, gluteal and thigh muscle size was positively correlated with proximal femur volumetric BMD [17]. Little is known about the correlation between BMD at the proximal femur and anatomical assessment of muscle and subcutaneous adipose tissue in older women with HFs. The objective of this study was to investigate the associations between the size and density of muscle and SAT and BMD of proximal femur in elderly women with HF in different osteoporosis conditions and different fracture types. The aim is to address the gaps in understanding the potential relationship between impairment of muscle, SAT, and bone function in elderly female patients from a macroscopic viewpoint.

## Materials and methods

### Participants

A total of 1166 female patients with suspected HFs, admitted to our hospital emergency department of orthopaedic trauma between January 2020 and July 2022, were recruited for this study. In this institution, CT scans are performed routinely for female subjects with suspected or confirmed HFs. Based on the CT images, fracture types were classified as either femoral neck fracture (FNF) or intertrochanteric fracture (ITF) by an experienced radiologist (J. L.). CT scans had to be conducted for all patients within 48 h after injury. The exclusion criteria for HF patients included: 1. Less than 50 years old; 2. High-energy HF (fall from non-standing or sitting height); 3. Previous bilateral HF or pelvic fracture; 4. Diseases that lead to long-term limited activity, such as hip dysplasia and avascular necrosis of the femoral head; 5. Poor body position or poor image quality.

The study was approved by the ethics committee of Shanghai Sixth People’s Hospital. Informed consent was obtained from each participant.

### Computed tomography acquisition

Spiral CT imaging of the hip was performed for all study participants with a uCT 780 scanner (United Imaging Healthcare Co., Ltd., Shanghai, China). Scans were acquired in supine position from the top of the acetabulum to 3 cm below the lesser trochanter and included both legs. Scan parameters for all CT scans were 120 kVp, 185 mAs, 50 cm field of view, 512 × 512 matrix, 0.5 s tube rotation time, 1 mm reconstructed slice thickness, and a standard convolution kernel algorithm. Air correction was performed daily, followed by an air scan to measure air CT values (within a standard limit of ± 6 HU). External calibration took place weekly, and the bed height was determined using laser alignment, referencing three crosses on the quality assurance body mold.

For cross-calibration of muscle attenuation in this CT scanner, HU values of the water equivalent material of the European Spine Phantom were measured.

### Muscle and SAT parameters assessment

CSA and density of the following muscle or muscle groups and MSAT were measured on 1 slice each. In subjects, the non-fractured hip was analysed. Within the hip, we analyzed the gluteus maximus muscle (G.MaxM) at the level of the greater trochanter and the gluteus medius and minimus muscles (G.Med/MinM) at the level of S3. In the thigh, analysis included all mid-thigh muscles (MM) located 3 cm below the lesser trochanter of the femur, along with the surrounding MSAT (Fig. 1).

SliceOmatic software (version 5.0; Tomovision, Montreal, Canada) was used for analysis. The ‘Region Growing’ tool was utilized to semi-automatically segment muscle and MSAT regions, adhering to preset HU intensity thresholds of − 29 to 150 HU for muscle and − 190 to – 30 HU for MSAT, respectively [18]. CSA and density values for muscle and MSAT in the regions of interest were automatically computed. All measurements were carried out by the same radiologist (Yt. L.), who had received prior training in CT imaging under the guidance of an expert radiologist (J. L.). For training, a sample of about 25 images had been analysed together with the expert prior to the beginning of the measurement study.

### Bone mineral density

Areal BMD (aBMD, g/cm2) of the femoral neck (FN), trochanter (TR), intertrochanter (IT), and total hip (TH) was calculated from the CT scans using the CTXA technique (Version 4.2.3, Mindways Inc.). The volume BMD (vBMD) at the corresponding sites was calculated by using Mindways QCT Pro BMD analysis Software (Mindways Software Inc., Austin, TX, USA). After image segmentation and manipulation of proximal femur rotation, a two-dimensional projection image was generated from the three-dimensional CT dataset [19]. The details have been described in previous research [20]. Osteoporosis was defined as a T-score of < − 2.5 according to the World Health Organization guideline. CTXA aBMD values of the total femur and FN are equivalent to DXA aBMD values [21]. In this context, we utilized the TH T-score to differentiate whether participants had osteoporosis.

### Statistical analyses

All statistical analyses were conducted using MedCalc (version 15.2.2, MedCalc Software bvba) and IBM SPSS (version 22.0, IBM). Continuous variables were described as mean ± standard deviation (SD). To assess differences between HFs within our non-normally distributed population, the Mann–Whitney *U* test was employed. Multivariable linear regression models investigated the relationship between BMD and both muscle and SAT, adjusting for age and BMI. Differences were considered significant at P < 0.05.

## Results

### Study cohort characteristics

After review of medical records and CT images, 505 participants (from 1166) were excluded from the HF groups. These exclusions included 92 participants younger than 50, 203 individuals with high-energy HF, 48 with prior bilateral hip or pelvic fractures, 50 with long-term diseases causing limited mobility, and 112 with inadequate image quality (i.e. motion artefacts). After exclusion, the study cohort included 661 participants: 440 subjects with FNF and 221 subjects with ITF. The characteristics of the study cohort are shown in Table 1.

**Table 1:** General characteristics of study participants

Characteristics	FNF	ITF	P-value^a^
	(n = 440)	(n = 221)	
Age (years)	72.75 ± 10.78	80.73 ± 10.24	< 0.001
Weight (kg)	56.64 ± 8.76	55.64 ± 9.21	0.172
Height (cm)	158.79 ± 5.02	156.72 ± 5.38	< 0.001
BMI (kg/m^2^)	22.43 ± 3.09	22.61 ± 3.35	0.686
T-score < − 2.5, % (n)	43.86 (193)	83.26 (184)	–
G.MaxM area (cm^2^)	33.23 ± 6.54	30.44 ± 6.60	< 0.001
G.MaxM density (HU)	32.84 ± 7.19	27.90 ± 8.34	< 0.001
G.Med/MinM area (cm^2^)	29.49 ± 6.13	27.65 ± 5.84	< 0.001
G.Med/MinM density (HU)	38.36 ± 6.30	33.53 ± 7.48	< 0.001
MM area (cm^2^)	84.23 ± 15.77	78.07 ± 14.96	< 0.001
MM density (HU)	40.94 ± 5.35	38.16 ± 5.60	< 0.001
MSAT area (cm^2^)	87.76 ± 25.60	83.76 ± 26.50	0.046
MSAT density (HU)	− 100.28 ± 4.97	-98.58 ± 6.97	0.008
TH aBMD (mg/cm^2^)	677.50 ± 106.94	553.09 ± 89.89	< 0.001
TH vBMD (mg/cm^3^)	250.54 ± 38.70	204.28 ± 33.36	< 0.001
FN aBMD (mg/cm^2^)	544.27 ± 94.91	513.76 ± 91.63	< 0.001
FN vBMD (mg/cm^3^)	247.11 ± 41.12	238.83 ± 42.67	0.014
TR aBMD (mg/cm^2^)	469.20 ± 100.32	378.67 ± 82.72	< 0.001
TR vBMD (mg/cm^3^)	163.98 ± 34.30	135.53 ± 27.71	< 0.001
IT aBMD (mg/cm^2^)	820.73 ± 126.35	677.69 ± 109.86	< 0.001
IT vBMD (mg/cm^3^)	300.04 ± 46.80	241.97 ± 41.42	< 0.001

*TH* total hip, *FNF* femoral neck fracture,* ITF* intertrochanteric fracture, *BMI* body mass index, *G.MaxM* gluteus maximus muscle, *G.Med/MinM*, gluteus medius and minimus muscle, *MM* midthigh muscle, *MSAT* midthigh subcutaneous adipose tissue, *BMD* bone mineral density, *aBMD* areal bone mineral density, *vBMD* volumetric bone mineral density, *TH* total hip, *FN* femoral neck, *TR* trochanter, *IT* intertrochanter, ^a^P-values were obtained for fracture risk using two-sample Wilcoxon tests for continuous variables

Compared to the ITF group, the FNF participants had higher height, G.MaxM area, G.MaxM density, G.Med/MinM area, G.Med/MinM density, MM area, MM density, MSAT area, and all BMD parameters (P < 0.05). Conversely, the age and MSAT density of the FNF participants was lower than that of the ITF group (P < 0.05).

### Total hip

Table 2 presents the adjusted B and 95% confidence intervals (CIs) for total hip BMD, related to continuous muscle and MSAT indexes, for each standard deviation (SD) increase, as determined by multivariable linear regression models analysis. After adjusting for other covariates, both TH aBMD and TH vBMD showed significant positive associations with G.Med/MinM density in the FNF group among participants with osteoporosis (P < 0.05). Simultaneously, within this group, 1.008 HU of G.MaxM density (95% CI, − 1.797–− 0.219; P = 0.013) decreased per SD increase of TH vBMD. In the FNF group without osteoporosis, there was a positive correlation between TH aBMD and G.MaxM density (B, 3.110; 95% CI, 0.305–5.916; P = 0.030), while TH vBMD exhibited a negative correlation with G.MaxM area (B, − 0.963; 95% CI, − 1.885–− 0.042; P = 0.041). However, no parameters related to BMD were found in the ITF group.

**Table 2:** Adjusted B and 95% CIs for sex-specific SD increase of total hip BMD with various muscle and subcutaneous adipose tissue indexes^a,b^

Variables	FNF				ITF				
	TH aBMD (mg/cm^2^)		TH vBMD (mg/cm^3^)			TH aBMD (mg/cm^2^)		TH vBMD (mg/cm^3^)	
	B (95% CI)	P-value	B (95% CI)	P-value		B (95% CI)	P-value	B (95% CI)	P-value
Osteoporosis									
G.MaxM area (cm^2^)	0.983 (− 0.567, 2.532)	0.212	0.209 (− 0.544, 0.961)	0.585		1.943 (− 0.160, 4.046)	0.070	0.841 (− 0.041, 1.724)	0.062
G.MaxM density (HU)	− 1.534 (− 3.159, 0.091)	0.064	− 1.008 (− 1.797, − 0.219)	0.013		0.807 (− 1.077, 2.690)	0.399	0.248 (− 0.543, 1.038)	0.537
G.Med/MinM area (cm^2^)	0.373 (-1.127, 1.872)	0.625	− 0.092 (− 0.820, 0.636)	0.803		0.042 (− 2.077, 2.162)	0.969	− 0.647 (-1.536, 0.243)	0.153
G.Med/MinM density (HU)	2.443 (0.609, 4.278)	0.009	1.433 (0.542, 2.324)	0.002		0.314 (− 1.919, 2.548)	0.782	0.180 (− 0.757, 1.118)	0.705
MM area (cm^2^)	0.109 (− 0.543, 0.762)	0.742	− 0.187 (− 0.504, 0.130)	0.246		0.632 (− 0.238, 1.503)	0.153	0.156 (− 0.210, 0.521)	0.401
MM density (HU)	0.312 (− 1.468, 2.092)	0.730	0.458 (− 0.407, 1.322)	0.298		1.276 (− 1.024, 3.576)	0.275	0.635 (− 0.331, 1.600)	0.196
MSAT area (cm^2^)	0.100 (− 0.232, 0.432)	0.552	− 0.039 (− 0.200, 0.122)	0.633		0.202 (− 0.285, 0.688)	0.414	0.050 (− 0.154, 0.254)	0.628
MSAT density (HU)	− 0.595 (− 1.971, 0.782)	0.395	− 0.194 (− 0.862, 0.475)	0.568		− 0.535 (-2.031, 0.962)	0.482	− 0.266 (-0.894, 0.362)	0.404
Nonosteoporosis									
G.MaxM area (cm^2^)	− 1.536 (− 3.863, 0.791)	0.195	− 0.963 (− 1.885, -0.042)	0.041		2.359 (− 0.767, 5.485)	0.133	1.010 (− 0.627, 2.646)	0.216
G.MaxM density (HU)	3.110 (0.305, 5.916)	0.030	0.559 (− 0.552, 1.670)	0.322		− 0.934 (-4.417, 2.549)	0.586	0.705 (− 1.118, 2.528)	0.434
G.Med/MinM area (cm^2^)	− 0.199 (− 2.339, 1.942)	0.855	− 0.437 (− 1.284, 0.411)	0.311		− 0.820 (-3.721, 2.080)	0.566	− 0.563 (− 2.081, 0.955)	0.453
G.Med/MinM density (HU)	− 1.081 (− 4.505, 2.343)	0.535	− 0.218 (− 1.573, 1.138)	0.752		2.722 (− 1.462, 6.905)	0.193	0.935 (− 1.255, 3.125)	0.388
MM area (cm^2^)	0.645 (− 0.372, 1.663)	0.213	− 0.036 (− 0.439, 0.367)	0.860		0.555 (− 0.790, 1.899)	0.404	− 0.136(-0.839, 0.568)	0.695
MM density (HU)	0.547 (− 2.510, 3.604)	0.725	0.689 (− 0.522, 1.899)	0.263		0.599 (− 4.087, 5.285)	0.795	− 0.231 (− 2.683, 2.222)	0.848
MSAT area (cm^2^)	− 0.133 (− 0.646, 0.380)	0.611	− 0.158 (− 0.361, 0.045)	0.126		− 0.066 (-0.691, 0.559)	0.829	0.052 (− 0.275, 0.380)	0.744
MSAT density (HU)	0.270 (− 2.182, 2.721)	0.829	− 0.388 (− 1.358, 0.583)	0.432		− 0.199 (− 2.375, 1.976)	0.852	0.123 (− 1.015, 1.262)	0.826

*SD* standard deviance, *BMD* bone mineral density, *FNF* femoral neck fracture, *ITF* intertrochanteric fracture, *TH* total hip; *aBMD* areal bone mineral density, *vBMD* volumetric bone mineral density, *CI* confidence interval; *G.MaxM* gluteus maximus muscle, *G.Med/MinM* gluteus medius and minimus muscle, *MM* midthigh muscle, *MSAT* midthigh subcutaneous adipose tissue

^a^Adjusted for age, body mass index (BMI)

^b^B for standard deviance increase of continuous muscle and subcutaneous adipose tissue variables

### Femoral neck

As shown in Table 3, in the FNF group, FN vBMD showed a positive correlation with G.MaxM density in the osteoporosis group, whereas it exhibited a negative correlation with G.MaxM density in the nonosteoporosis group. (Osteoporosis: B, − 1.546; 95% CI, − 2.567–− 0.524; P = 0.003 vs. nonosteoporosis: B, 1.349; 95% CI, 0.167–2.531; P = 0.025). FN aBMD was also significantly positive correlated with G.MaxM density in the absence of osteoporosis (B, 2.765; 95% CI, 0.242–5.287; P = 0.032). Furthermore, among participants with osteoporosis, 2.065 HU of G.Med/MinM density increased per SD increase of FN vBMD (95% CI, 0.912–3.217; P = 0.001).

**Table 3:** Adjusted B and 95% CIs for sex-specific SD increase of various muscle and subcutaneous adipose tissue indexes with femoral neck BMD^a,b^

Variables	FNF				ITF			
	FN aBMD (mg/cm^2^)		FN vBMD (mg/cm^3^)		FN aBMD (mg/cm^2^)		FN vBMD (mg/cm^3^)	
	B (95% CI)	P-value	B (95% CI)	P-value	B (95% CI)	P-value	B (95% CI)	P-value
Osteoporosis								
G.MaxM area (cm^2^)	1.358 (− 0.429, 3.145)	0.136	0.559 (− 0.415, 1.533)	0.259	1.113 (− 1.412, 3.639)	0.385	0.635 (− 0.661, 1.930)	0.335
G.MaxM density (HU)	− 1.397 (− 3.272, 0.478)	0.143	− 1.546 (− 2.567, -0.524)	0.003	− 0.817 (− 3.079, 1.445)	0.477	− 0.178 (− 1.338, 0.983)	0.763
G.Med/MinM area (cm^2^)	0.256 (− 1.473, 1.985)	0.771	- 0.913(− 1.855, 0.029)	0.057	− 0.885 (− 3.431, 1.661)	0.494	− 1.300 (− 2.606, 0.006)	0.051
G.Med/MinM density (HU)	1.950 (− 0.166, 4.066)	0.071	2.065 (0.912, 3.217)	0.001	1.281 (− 1.402, 3.963)	0.347	0.356 (− 1.020, 1.733)	0.610
MM area (cm^2^)	0.085 (− 0.668, 0.838)	0.824	0.004 (− 0.406, 0.414)	0.984	0.878 (− 0.168, 1.924)	0.099	0.385 (− 0.151, 0.921)	0.158
MM density (HU)	0.330 (− 1.723, 2.383)	0.751	0.380 (− 0.739, 1.498)	0.504	1.087 (− 1.676, 3.849)	0.439	1.311 (− 0.106, 2.728)	0.070
MSAT area (cm^2^)	0.148 (− 0.235, 0.532)	0.445	0.071 (− 0.137, 0.280)	0.501	0.265 (− 0.319, 0.850)	0.371	0.109 (− 0.191, 0.408)	0.476
MSAT density (HU)	− 0.116 (− 1.704, 1.472)	0.886	0.015 (− 0.850, 0.880)	0.973	− 0.374 (− 2.171, 1.423)	0.682	− 0.060 (− 0.982, 0.862)	0.897
Nonosteoporosis								
G.MaxM area (cm^2^)	− 0.542 (− 2.635, 1.551)	0.610	− 0.957 (− 1.938, 0.023)	0.056	− 1.555 (− 6.330, 3.221)	0.509	− 0.230 (− 2.533, 2.073)	0.839
G.MaxM density (HU)	2.765 (0.242, 5.287)	0.032	1.349 (0.167, 2.531)	0.025	− 6.078 (− 11.398, -0.757)	0.027	− 1.798 (− 4.364, 0.769)	0.162
G.Med/MinM area (cm^2^)	0.524 (− 1.401, 2.449)	0.593	− 0.080 (− 0.982, 0.821)	0.861	− 0.627 (− 5.058, 3.804)	0.774	− 0.903 (− 3.040, 1.234)	0.393
G.Med/MinM density (HU)	0.177 (− 2.902, 3.257)	0.910	− 0.066 (− 1.508, 1.377)	0.929	9.608 (3.216, 15.999)	0.005	3.472 (0.390, 6.555)	0.029
MM area (cm^2^)	-0.238 (− 1.154, 0.677)	0.608	− 0.113 (− 0.541, 0.316)	0.605	− 0.009 (− 2.063, 2.044)	0.993	− 0.268 (− 1.259, 0.722)	0.583
MM density (HU)	− 1.618 (− 4.368, 1.131)	0.247	− 0.554 (− 1.843, 0.734)	0.397	1.088 (− 6.070, 8.246)	0.757	− 0.183 (− 3.635, 3.269)	0.914
MSAT area (cm^2^)	0.060 (− 0.402, 0.521)	0.799	− 0.099 (− 0.316, 0.117)	0.366	− 0.476 (− 1.431, 0.479)	0.315	− 0.159 (− 0.620, 0.301)	0.483
MSAT density (HU)	1.167 (− 1.038, 3.371)	0.298	− 0.020 (− 1.053, 1.013)	0.969	0.128 (− 3.195, 3.452)	0.937	1.097 (− 0.506, 2.699)	0.171

*SD* standard deviance, *BMD* bone mineral density, *FNF* femoral neck fracture, *ITF* intertrochanteric fracture, *FN* femoral neck, *aBMD* areal bone mineral density, *vBMD* volumetric bone mineral density, *CI* confidence interval, *G.MaxM* gluteus maximus muscle, *G.Med/MinM* gluteus medius and minimus muscle, *MM *midthigh muscle, *MSAT* midthigh subcutaneous adipose tissue

^a^Adjusted for age, body mass index (BMI)

^b^B for standard deviance increase of continuous muscle and subcutaneous adipose tissue variables

In the ITF group, both FN aBMD and FN vBMD showed significant positive correlations with G.Med/MinM density in participants without osteoporosis (aBMD: B, 9.608; 95% CI, 3.216–15.999; P = 0.005 vs. vBMD: B, 3.472; 95% CI, 0.390–6.555; P = 0.029). In addition, among participants without osteoporosis, 6.078 HU of G.MaxM density decreased per SD increase of FN aBMD (95% CI, -11.398–-0.757; P = 0.027). However, no parameters associated with BMD were found in the group with osteoporosis.

### Trochanter

The adjusted results of multivariable linear regression models for the associations between TR BMD and both muscle and SAT indexes are presented in Table 4. In the FNF group, among participants with osteoporosis, TR aBMD (B, 2.786; 95% CI, 0.386–5.187; P = 0.023) and TR vBMD (B, 1.303; 95% CI, 0.466–2.140; P = 0.002) were significantly positive correlated with G.Med/MinM density. Furthermore, there was a positive correlation between TR aBMD and MM area (B, 0.942; 95% CI, 0.088–1.795; P = 0.031), as well as between TR aBMD and MSAT area (B, 0.453; 95% CI, 0.018–0.888; P = 0.041). When participants did not have osteoporosis, TR aBMD (B, − 2.408; 95% CI, − 4.561–− 0.255; P = 0.029) and TR vBMD (B, − 1.225; 95% CI, − 2.028–− 0.422; P = 0.003) were significantly negative correlated with G.MaxM area; 3.781 HU of G.MaxM density increased per SD increase of TR aBMD (95% CI, 1.186–6.376; P = 0.004). However, no parameters related to BMD were found in the ITF group.

**Table 4:** Adjusted B and 95% CIs for sex-specific SD increase of various muscle and subcutaneous adipose tissue indexes with trochanter BMD^a,b^

Variables	FNF										ITF							
	TR aBMD (mg/cm^2^)		TR vBMD (mg/cm^3^)					TR aBMD (mg/cm^2^)								TR vBMD (mg/cm^3^)		
	B (95% CI)	P-value	B (95% CI)	P-value				B (95% CI)					P-value			B (95% CI)		P-value
Osteoporosis																		
G.MaxM area (cm^2^)	0.277 (-1.751, 2.304)	0.788	-0.022(-0.729, 0.686)			0.952	1.795(-0.198, 3.787)			0.077		0.517 (-0.168, 1.201)			0.138			
G.MaxM density (HU)	0.275 (-1.852, 2.402)	0.799	-0.215(-0.956, 0.527)			0.569	0.883(-0.901, 2.668)			0.330		0.207 (-0.406, 0.821)			0.505			
G.Med/MinM area (cm^2^)	-0.041 (-2.003, 1.921)	0.967	-0.113(-0.798, 0.571)			0.744	-0.111(-2.120, 1.897)			0.913		-0.528 (-1.219, 0.162)			0.133			
G.Med/MinM density (HU)	2.786 (0.386, 5.187)	0.023	1.303(0.466, 2.140)			0.002	0.253(-1.864, 2.369)			0.814		0.357 (-0.370, 1.084)			0.334			
MM area (cm^2^)	0.942(0.088, 1.795)	0.031	0.214(-0.083, 0.512)			0.157	0.320(-0.505, 1.145)			0.445		0.140 (-0.143, 0.424)			0.330			
MM density (HU)	-0.472 (-2.801, 1.857)	0.690	0.104(-0.708, 0.916)			0.800	1.504(-0.675, 3.684)			0.175		0.368 (-0.380, 1.117)			0.333			
MSAT area (cm^2^)	0.453 (0.018, 0.888)	0.041	0.112(-0.040, 0.263)			0.147	0.415(-0.046, 0.876)			0.077		0.108(-0.050, 0.267)			0.179			
MSAT density (HU)	0.509 (-1.293, 2.310)	0.578	0.265(-0.363, 0.893)			0.406	-0.762(-2.179, 0.656)			0.290		-0.333(-0.820, 0.154)			0.179			
**N**onosteoporosis																		
G.MaxM area (cm^2^)	-2.408 (-4.561, -0.255)	0.029	-1.225(-2.028, -0.422)		0.003				3.600(-1.710, 8.909)		0.175			1.509(-0.436, 3.455)			0.123	
G.MaxM density (HU)	3.781 (1.186, 6.376)	0.004	0.642(-0.326, 1.610)		0.193				0.917(-4.999, 6.833)		0.753			0.276(-1.891, 2.444)			0.795	
G.Med/MinM area (cm^2^)	1.171 (-0.809, 3.151)	0.245	-0.002(-0.740, 0.737)		0.996				-0.940(-5.866, 3.987)		0.698			-0.607(-2.412, 1.198)			0.495	
G.Med/MinM density (HU)	-1.343 (-4.510, 1.825)	0.405	0.151(-1.030, 1.333)		0.801				1.267(-5.839, 8.373)		0.717			0.420(-2.184, 3.023)			0.743	
MM area (cm^2^)	0.514 (-0.427, 1.456)	0.283	0.171(-0.180, 0.522)		0.338				-0.021(-2.304, 2.262)		0.985			-0.222(-1.058, 0.615)			0.590	
MM density (HU)	-0.473 (-3.301, 2.356)	0.742	0.120(-0.935, 1.175)		0.823				0.644(-7.315, 8.603)		0.869			0.028(-2.888, 2.944)			0.984	
MSAT area (cm^2^)	-0.246 (-0.721, 0.229)	0.308	-0.145(-0.322, 0.032)		0.107				0.141(-0.920, 1.203)		0.786			0.047(-0.342, 0.436)			0.807	
MSAT density (HU)	0.092 (-2.176, 2.360)	0.936	-0.162(-1.008, 0.684)		0.707				-0.084(-3.779, 3.611)		0.963			0.091(-1.263, 1.445)			0.891	

*SD* standard deviance, *BMD* bone mineral density, *FNF* femoral neck fracture, *ITF* intertrochanteric fracture, *TR* trochanter, *aBMD* areal bone mineral density, *vBMD* volumetric bone mineral density, *CI* confidence interval, *G.MaxM* gluteus maximus muscle, *G.Med/MinM* gluteus medius and minimus muscle, *MM* midthigh muscle, *MSAT*, midthigh subcutaneous adipose tissue

^a^Adjusted for age, body mass index (BMI)

^b^B for standard deviance increase of continuous muscle and subcutaneous adipose tissue variables

### Trochanter

As shown in Table 5, in the FNF group, among participants with osteoporosis, IT aBMD and IT vBMD exhibited negative correlations with G.MaxM density (aBMD: B, − 2.659; 95% CI, − 4.870–− 0.448; P = 0.019 vs. vBMD: B, − 1.630; 95% CI, − 2.683–− 0.577; P = 0.003), whereas they displayed positive correlations with G.Med/MinM density (aBMD: B, 3.074; 95% CI, 0.579–5.569; P = 0.016 vs. vBMD: B, 1.888; 95% CI, 0.699–3.076; P = 0.002). Conversely, in the absence of osteoporosis, there was no significant correlation between these variables.

**Table 5:** Adjusted B and 95% CIs for sex-specific SD increase of various muscle and subcutaneous adipose tissue indexes with intertrochanter BMD^a,b^

Variables	FNF						ITF		
	IT aBMD (mg/cm^2^)		IT vBMD (mg/cm^3^)		IT aBMD (mg/cm^2^)			IT vBMD (mg/cm^3^)	
	B (95% CI)	P-value	B (95% CI)	P-value	B (95% CI)	P-value		B (95% CI)	P-value
Osteoporosis									
G.MaxM area (cm^2^)	1.823 (− 0.285, 3.931)	0.090	0.565(− 0.439, 1.569)	0.268	2.385(− 0.377, 5.147)	0.090		1.344(0.164, 2.524)	0.026
G.MaxM density (HU)	− 2.659 (− 4.870, -0.448)	0.019	− 1.630(− 2.683, -0.577)	0.003	0.891(− 1.583, 3.365)	0.478		0.369(− 0.687, 1.426)	0.491
G.Med/MinM area (cm^2^)	0.228 (− 1.812, 2.268)	0.826	− 0.141(− 1.113, 0.830)	0.774	0.097(− 2.687, 2.882)	0.945		− 0.557(− 1.746, 0.632)	0.357
G.Med/MinM density (HU)	3.074 (0.579, 5.569)	0.016	1.888(0.699, 3.076)	0.002	1.004(− 1.930, 3.938)	0.500		0.037(− 1.216, 1.290)	0.954
MM area (cm^2^)	0.147 (− 0.741, 1.035)	0.744	− 0.361(− 0.784, 0.062)	0.094	0.933(− 0.211, 2.077)	0.109		0.105(− 0.383, 0.594)	0.671
MM density (HU)	0.236 (− 2.186, 2.657)	0.848	0.552(− 0.602, 1.705)	0.346	0.457(− 2.565, 3.478)	0.766		0.713(− 0.577, 2.004)	0.277
MSAT area (cm^2^)	0.058 (− 0.394, 0.510)	0.801	− 0.072(− 0.288, 0.143)	0.507	0.067(− 0.573, 0.706)	0.837		0.020(− 0.253, 0.293)	0.887
MSAT density (HU)	-0.524 (− 2.397, 1.349)	0.582	− 0.338(− 1.230, 0.554)	0.456	− 0.164(-2.129, 1.802)	0.870		-0.164(− 1.004, 0.676)	0.700
Nonosteoporosis									
G.MaxM area (cm^2^)	-1.002 (-3.877, 1.873)	0.493	− 0.684 (− 1.875, 0.507)	0.259	2.636 (− 1.526, 6.797)	0.204		0.695 (− 1.625, 3.015)	0.543
G.MaxM density (HU)	3.398 (-0.068, 6.864)	0.055	0.453 (− 0.983, 1.889)	0.535	− 1.648 (-6.285, 2.989)	0.472		1.554 (− 1.031, 4.139)	0.228
G.Med/MinM area (cm^2^)	-0.708 (-3.352, 1.937)	0.599	− 0.482 (− 1.577, 0.614)	0.387	0.125 (− 3.737, 3.986)	0.948		0.067 (− 2.085, 2.220)	0.949
G.Med/MinM density (HU)	-0.509 (-4.739, 3.721)	0.813	− 0.119 (− 1.872, 1.634)	0.894	4.133 (− 1.436, 9.703)	0.139		1.206 (− 1.899, 4.311)	0.432
MM area (cm^2^)	0.815 (-0.442, 2.072)	0.203	− 0.153 (− 0.674, 0.368)	0.563	0.753 (− 1.036, 2.543)	0.395		− 0.068 (− 1.066, 0.929)	0.889
MM density (HU)	-0.031 (-3.808, 3.747)	0.987	0.761 (− 0.804, 2.326)	0.339	− 1.035 (-7.273, 5.204)	0.736		− 0.668 (− 4.146, 2.809)	0.696
MSAT area (cm^2^)	-0.089 (-0.723, 0.544)	0.781	− 0.183 (− 0.446, 0.080)	0.171	0.041 (− 0.791, 0.873)	0.921		0.152 (− 0.312, 0.615)	0.508
MSAT density (HU)	0.396 (-2.633, 3.424)	0.797	− 0.623 (− 1.877, 0.632)	0.329	0.257 (− 2.639, 3.154)	0.857		0.115 (− 1.500, 1.729)	0.885

*SD* standard deviance, *BMD* bone mineral density, *FNF* femoral neck fracture, *ITF* intertrochanteric fracture, *IT* intertrochanter, *aBMD* areal bone mineral density, *vBMD* volumetric bone mineral density, *CI* confidence interval *G.MaxM* gluteus maximus muscle, *G.Med/MinM* gluteus medius and minimus muscle, *MM* midthigh muscle, *MSAT* midthigh subcutaneous adipose tissue

^a^Adjusted for age, body mass index (BMI)

^b^B for standard deviance increase of continuous muscle and subcutaneous adipose tissue variables

In the ITF group, 1.344 cm^2^ of G.MaxM area increased per SD increase of IT vBMD (95% CI, 0.164–2.524; P = 0.026), while other parameters did not show significant correlations.

## Discussion

The new finding of this study is that in older women with HFs, density but not cross-sectional area of the G.Med/MinM were associated with BMD parameters of the proximal femur, especially in the FNF group with osteoporosis. This suggests that muscle density in this region is more important than muscle size for localized bone. We also found that in the FNF group, G.MaxM density was negatively associated with the BMD parameters of the proximal femur in individuals with osteoporosis, while they were positively associated with the absence of osteoporosis. However, in the ITF group, we did not observe a similar correlation. This suggests that osteoporosis may influence the relationship between G.MaxM density and proximal femur BMD in elderly women with FNF.

A close relationship exists between bone and muscle from embryogenesis, through growth and development, and into aging. Throughout life, bone and muscle integrate with each other and work physically and biochemically as one unit [10]. Bone and muscle experience organogenesis through tightly orchestrated gene activation and inactivation so that bone and muscle develop synchronously [22]. Skeletal muscles attach to bone and contraction is responsible for movement of the bone and therefore locomotion by the organism. Muscle is attached to bone close to the axes of motion, generating small lever arms requiring large muscle forces to produce the motion-required torque. Forces generated by muscle are the source of mechanical loading that generates the strain in bone [10]. As commonly understood, ITFs typically result from sudden twisting of the lower limbs, strong adduction or abduction during falls, or direct external impact, often leading to comminuted fractures due to local osteoporosis and fragility. ITF are categorized as extra-capsular fractures, and the blood supply to the intertrochanteric region is relatively rich. Conversely, FNFs primarily manifest as spiral or oblique fractures resulting from external rotational forces, representing capsular fractures that are more susceptible to blood supply compromise. Aitken [23] reported that ITFs were seen more commonly in women with severe osteoporosis (p < 0.005), whereas FNFs predominated in those who did not have osteoporosis, consistent with our findings. Based on this, we speculate that osteoporosis and blood supply differences may contribute to variations in association patterns between the FNF and ITF groups.

Studies [24–26], have shown that an increase in the thickness of the greater trochanter of the femur, or the soft tissue near the thigh, reduces the risk of HFs by decreasing the external force exerted on the femur during lateral falls. Bouxsein, M. L. et al. [24] found that in postmenopausal women, there was an 82% increase in fracture risk for every one standard deviation reduction in soft tissue thickness of the greater trochanteric femur. In the study by Yin, L. et al. [17], it was observed that an increase in G.MaxM area was associated with higher TR cortical vBMD in both sexes. This suggests that the impact of soft tissue thickness on HF risk may not solely involve attenuating external forces but also strengthening the neighboring cortical bone. However, our study’s results differed slightly from those of Yin, L. et al., who found that only a few BMD parameters were negatively correlated with G.MaxM area in FNF without osteoporosis. The discrepancy may be attributed to differences in the populations analyzed. The former study focused on healthy elderly individuals, whereas our analysis involved elderly women with HFs. Moreover, we observed that in the FNF group with osteoporosis, after adjusting for covariates, there was an association between an increase in G.Med/MinM density and higher BMD, indicating that an increase in gluteal muscle density might also mitigate the risk of HFs. The importance of G.Med/MinM density was also demonstrated in a recent study [27], which proved that density of the G.Med/MinM was an aBMD independent predictor of the risk of second HF.

The gluteus muscle is widely regarded as one of the strongest muscles in the human body, and the G.Med/MinM is often referred to as the ‘rotator cuff of the hip’ [28]. G.Med/MinM attaches to the greater trochanter of the femur and serves as the primary abductors and rotators of the hip, playing a crucial role in maintaining balance during normal gait [29, 30]. Muscle density assessed via CT is quantified by the X-ray attenuation of the muscle, reported as the CT value (HU), which indicates the amount of adipose tissue within the muscle. An increase in adipose tissue accumulation within the muscle decreases the HU value, but it does not affect the muscle area [31]. In this regard, the explanation is that changes in muscle density precede hip fractures and could potentially serve as a long-term predictor of the fragility leading to HFs [32]. Substantial decreases in skeletal muscle function with ageing can occur with only minimal loss of skeletal muscle mass [33, 34]. This discrepancy may be partially related to the presence of fatty infiltration, which is 1 aspect of muscle quality (muscle density). Depletion of skeletal muscle mass also seems to trigger bone loss due to the unloading of bone [10]. The preceding explanation partially elucidates why muscle density holds greater significance than size in the correlation analysis between muscle parameters and BMD parameters.

Adipocytes are important sources of estrogen production in postmenopausal women, and estrogen is known to inhibit bone resorption by osteoclasts [12]. It has been proposed that increases in adipose tissue, with increasing BMI in postmenopausal women, results in increased estrogen production, osteoclast suppression, and a resultant increase in bone mass [35]. A study [12] revealed that fat mass is inversely correlated with bone mass genetically, environmentally, and phenotypically, when the mechanical loading effects of body weight on bone mass are controlled. Studies utilizing dual-energy X-ray absorptiometry [36, 37] have indicated that increasing fat thickness may spuriously decrease total body BMD. In order to effectively attenuate the impact of a typical fall state in the older people, there has been a suggestion to introduce a pad, known as a hip protector, around the buttocks, akin to augmenting the thickness of SAT [38]. A decrease in SAT adjacent to the femoral trochanter could lead to heightened pressure on the buttocks in the event of a fall [39]. In both males and females, a 4% increase in fracture risk for every 1-mm decrease in mid-thigh subcutaneous fat [40]. Regrettably, in our study, neither the area nor the density of MSAT showed a significant correlation with hip BMD parameters. Furthermore, another study [41] indicated that lumbar BMD did not correlate with peripheral SAT levels in Chinese women.

Our study has some limitations. First, this study was a cross-sectional study, so it was unable to investigate the impact of muscle size and density on BMD changes at the organ, cellular, and molecular levels. Second, muscle and SAT can only be assessed at a single slice, precluding the possibility of conducting a 3D analysis of entire muscle structures or muscle groups and their corresponding levels of SAT. Lastly, our study exclusively focused on women aged over 50, predominantly postmenopausal, and therefore, the results cannot be extrapolated to older men.

## Conclusions

Our studies show that in older women with HFs, density but not cross-sectional area of the G.Med/MinM were associated with BMD parameters of the proximal femur, especially in the FNF group with osteoporosis. In addition, in the FNF group, G.MaxM density was negatively associated with the BMD parameters of the proximal femur in individuals with osteoporosis, while they were positively associated with the absence of osteoporosis. This suggests that osteoporosis may influence the relationship between G.MaxM density and proximal femur BMD in elderly women with FNF. Therefore, hip muscle density might represent a more clinically significant focus for osteoporosis treatment and HF prevention.

### Supplementary Information

Below is the link to the electronic supplementary material.

Supplementary file1 (DOCX 666 KB)
