# Supplementary material for: Relationship between muscle and subcutaneous adipose tissue size and density and proximal femur bone in elderly women with hip fracture
Source: Aging Clin Exp Res. 2024 Jun 11;36(1):130. doi: 10.1007/s40520-024-02782-y (PMC11166751; doi:10.1007/s40520-024-02782-y)
Supplement: Supplementary file 1 — Supplementary file1 (DOCX 666 KB) [file 40520_2024_2782_MOESM1_ESM.docx]

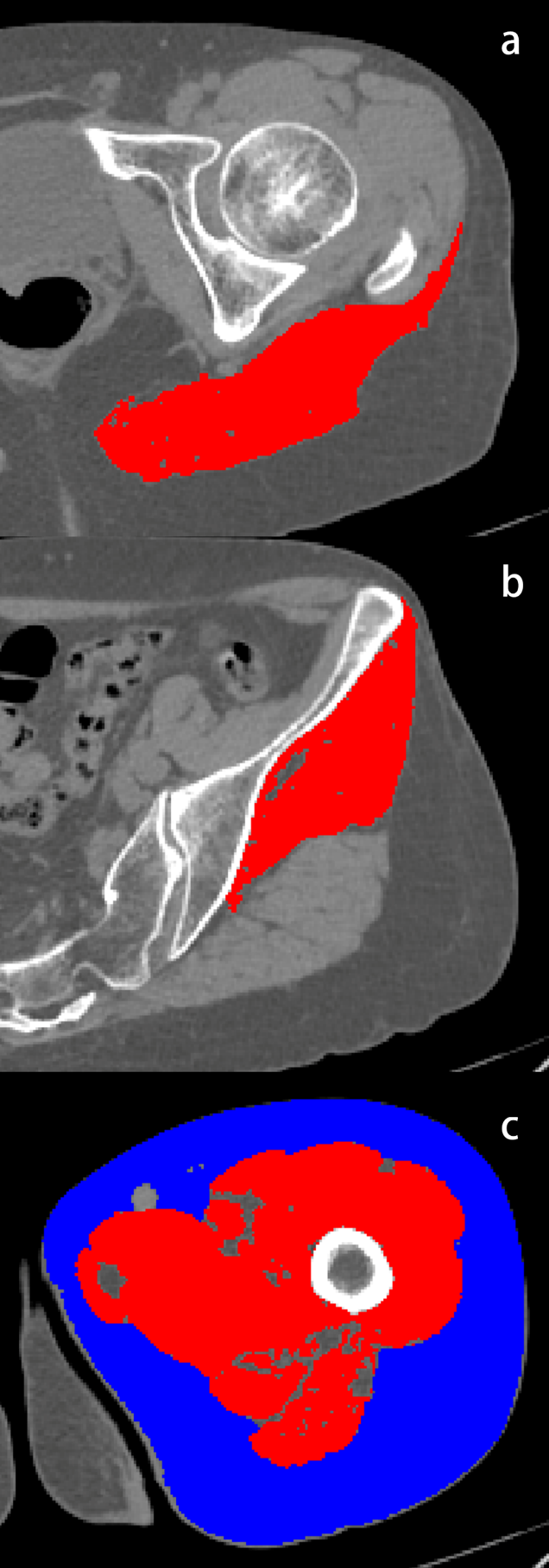


eFigure 1 The cross-sectional level of muscle and subcutaneous adipose tissue measurement (A–C). (A) Measurement of the gluteus maximus at the level of the greater trochanter of the femur; (B) Measurement of the gluteus medius and minimus muscle at the 3rd sacral (S3) level; (C) Measurement of the mid-thigh muscle group and subcutaneous adipose tissue. Muscle and subcutaneous adipose tissue areas are represented by areas highlighted in red and blue, respectively.
